# Immune cell puzzle COVID-19: how do SARS-CoV infections contribute to psychiatric diseases?

**DOI:** 10.1007/s00406-020-01179-y

**Published:** 2020-08-04

**Authors:** Matthias Jung, Dan Rujescu

**Affiliations:** Martin-Luther-University Halle-Wittenberg Clinic and Polyclinic for Psychiatry, Psychotherapy and Psychosomatic Medicine, Halle/Saale, Germany

Neuropsychiatric diseases such as Alzheimer’s disease (AD) and other neurodegenerative disorders are most often associated with ageing. The number of elderly people is growing in our society leading to an increasingly growing number of AD patients. Accordingly, AD is among the major causes of suffering and death in the USA, in Europe, and in other countries worldwide. In the USA, it has been estimated that about 5.8 million people will suffer from AD in 2020 [1]. AD pathology seems to have a variety of cellular and molecular disease mechanisms. Even though a large number of clinical trials have been conducted and are currently ongoing, the translation from bench to bedside remains difficult. This is highlighted by the very low number of drugs launched for the market within the last decade. The need for additional treatment strategies encouraged the scientific community to broaden the field of AD research, especially in the context of molecular ageing. There is a growing interest in age-related differences during healthy ageing and accelerated ageing, especially regarding the immune system. Findings pointing to the immune system were obtained from research in the field of human genetics, which revealed that a large number of AD-associated DNA variations occur in genes transcribed in immune cells of the central nervous system named microglia [2]. These DNA variations include single-nucleotide polymorphisms (SNPs), and functional mutations that confer a risk for AD. DNA variations influence AD onset, progression, and AD treatment. In AD, the disturbed immune system leads to proinflammatory processes accelerating neuronal loss and disease progression. This kind of immunopathology is in focus for AD, but has been also recently described in endothelial cells and peripheral blood mononuclear cells in the context of COVID-19 [3].

Coronavirus disease 2019 (COVID-19) is a worldwide ongoing pandemic first identified in December 2019 resulting in about 700,000 death until July 2020. COVID-19 is caused by the infection of the lung with the severe acute respiratory syndrome coronavirus 2 (SARS-CoV-2) predominantly affecting older people and people with preexisting medical conditions. These patients have a higher risk to develop devastating clinical symptoms including lymphocytopenia and its associated cytokine storm [4]. Compared to moderate COVID-19 cases, critical cases exhibited stronger interactions between epithelial and immune cells in the lung, which was recently demonstrated by a large interdisciplinary study under participation of Christian Drosten at the Charite in Berlin, Germany [5]. In addition, T-cell weakness is related to ageing. This suggests an age-dependent disturbed immune response leading to chronic inflammation and a higher risk for old age people to suffer from devastating clinical symptoms [6]. Latest insights from COVID-19 research provide evidence that all organs of the human body can be infected and disturbed including the central nervous system. SARS-Cov-2 was found in post-mortem brains of COVID-19 patients. Many patients lost their taste and smell for a duration of up to 2 weeks after infection. Furthermore, infection of brain cells may be involved in the development of the acute respiratory distress syndrome (ARDS). It is discussed whether SARS-CoV-2 spread via a synapse-connected route to the medullary cardiorespiratory center from the mechanoreceptors and chemoreceptors in the lung and lower respiratory airways [7]. The high diversity of SARS-CoV-2 interactions in the human body and its impact on the central nervous system can be described as a COVID-19 immune cell puzzle with many unknown puzzle pieces, especially from the central nervous system.

One of the most urgent questions in the field of COVID-19 research is how ageing and/or disturbed immunity contributes to devastating clinical symptoms. These questions can be raised similarly for AD. It appears also plausible that there is a negative impact from COVID-19 on AD, because pneumonia is the leading cause to death in the elderly with dementia [8].

The detailed analysis of molecular and cellular disease mechanisms in the human brain related to the above raised questions requires the application of human cellular two-dimensional and organotypic three-dimensional in vitro models mimicking the tissue of the human brain. Furthermore, these cultures can be generated from old age AD patients using induced pluripotent stem cells (iPS cells). Rodents are broadly applied for disease modeling, but notably these animals poorly mimic the complex pathology of COVID-19 and AD in humans, especially in the context of ageing. Therefore, human in vitro models hold a great promise to improve our understanding of COVID-19 and its impact on AD. This is necessary for further advance aiming at the development of improved and patient-tailored COVID-19 therapies for AD.
